# Do Online Gambling Products Require Traditional Therapy for Gambling Disorder to Change? Evidence from Focus Group Interviews with Mental Health Professionals Treating Online Gamblers

**DOI:** 10.1007/s10899-021-10064-9

**Published:** 2021-10-16

**Authors:** Hibai Lopez-Gonzalez, Susana Jimenez-Murcia, Alicia Rius-Buitrago, Mark D. Griffiths

**Affiliations:** 1 Department of Library, Information Science, and Communication, University of Barcelona, Melcior de Palau 140, 08014 Barcelona, Spain; 2 Department of Psychiatry, University Hospital of Bellvitge-IDIBELL, Barcelona, Spain; 3 Ciber Fisiopatologia Obesidad y Nutrición (CIBERobn), Instituto Salud Carlos III, Madrid, Spain; 4 Department of Clinical Sciences, School of Medicine and Health Sciences, University of Barcelona, Barcelona, Spain; 5 Instituto Mujeres y Cooperación, Madrid, Spain; 6 International Gaming Research Unit, Psychology Department, Nottingham Trent University, Nottingham, UK

**Keywords:** Gambling disorder, Online gambling, CBT, Mental health professionals, Gambling preferences, Strategic gambling

## Abstract

Online gambling has significantly altered the situational and structural characteristics of gambling products, to the extent that online gamblers might be substantially different from traditional offline gamblers. A growing body of literature has identified the evolving features of online gambling and the individuals who engage in it. However, beyond understanding the individual characteristics of this subgroup, relatively less effort has been made to examine whether existing cognitive-behavioural therapy (CBT) approaches are still entirely relevant for online problem gamblers, or whether changes are needed to adapt according to gambling mode of access. To understand what kind of challenges online gambling poses to mental health professionals dealing with disordered gamblers, four focus groups comprising 28 Spanish participants were carried out. All the treatment providers had ongoing experience with online gamblers undergoing treatment, and included clinical psychologists, mental health social workers, and a medical doctor. The data were examined using thematic analysis. The analysis identified five main themes that characterised online gamblers: (1) being of younger age, (2) lack of conflicts at home and at work/educational centre, rarely presenting violent or aggressive behaviour, (3) gambling disorder only being identified by overdue debt, (4) co-occurring conditions with technology-related abuse rather than other substance-related addictions, and (5) skill-based gambling. The study highlights mental health workers’ perceived insecurities about how to best treat online gamblers, and discusses the specific characteristics that CBT for gambling disorder might need to incorporate to adjust for this particular group of gamblers.

**Supplementary Information:**

The online version contains supplementary material available at 10.1007/s10899-021-10064-9.

## Introduction

In parallel to the growth of online gambling participation worldwide (Gainsbury et al., 2015; Wood & Williams, 2009), the number of treatment-seeking gamblers who prefer to gamble online is rising (Hing et al., 2015; Sharman et al., 2019). While evidence indicates that offline gamblers are overrepresented in clinical settings (Moragas et al., 2015) as well as helplines assisting problem gamblers (Ledgerwood et al., 2012), a small number of jurisdictions (e.g., Sweden) have reported that online gamblers seeking help have surpassed offline gamblers for the first time (Håkansson et al., 2017).

The COVID-19 pandemic has provided an additional impulse for online gambling as an alternative to the restrictions applied to land-based gambling venues (Brodeur et al., 2021). Many individuals have seen their free time (and time at home) increased during the lockdowns and some have resorted to online gambling as a means to cope with boredom, social isolation, and psychological distress (Wardle et al., 2021). Although the actual impact of the COVID-19 outbreak on problem gambling remains to be determined (Gainsbury et al., 2020; Hodgins & Stevens, 2021; Zamboni et al., 2021), the evidence suggests that there are more active online gamblers than before the pandemic (Auer & Griffiths, 2021). In Spain, where the current study was carried out, registered online gamblers increased by 8.36% from 2019 to 2020 (Dirección General de Ordenación del Juego, 2021).

Online gamblers exhibit distinctive psychological, behavioural, and sociological features that make them different in some aspects to traditional gamblers undergoing treatment for gambling disorder. Online problem gamblers, as compared to offline gamblers, have been reported more likely to be males, younger, with lower psychological distress, experiencing problems with sports and race betting, and less likely of seeking for help (Hing et al., 2015; Wardle et al., 2011). Accumulated debt for online gambler also tend to be larger than for offline gamblers (Estévez et al., 2017). A confounding aspect of online gambling has to do with the skill versus chance axis that traverses it. To put it simply, games that incorporate skill-based elements (also called strategic games—e.g., online sports betting and online poker) account for a larger proportion of the online gambling market compared to their weight within the traditional offline gambling market (European Commission, 2011; European Gaming & Betting Association, 2016; Paddy Power Betfair plc, 2019; William Hill PLC, 2019).

This shift towards greater online gambling has multiple ramifications for the treatment of online gamblers. More strategic gambling forms appear to be associated with a greater illusion of control (Toneatto et al., 1997), a result which has been later replicated and extended to both pathological and non-pathological gamblers (Myrseth et al., 2010). Strategic gamblers also tend to be male, younger, more sensation-seeking, although their cognitive function did not differ from non-strategic gamblers (Bonnaire et al., 2017; Stevens & Young, 2010). Similarly, other research has indicated that strategic gamblers tend to be more analytical and less intuitive than non-strategic ones (Mouneyrac et al., 2018).

In a recent study in a large clinical setting, strategic problem gamblers were more likely than non-strategic problem gamblers to be male, younger, with higher education, higher socioeconomic status, higher progression and earlier onset of gambling disorder, lower comorbid risk, higher novelty seeking, and higher risk of relapse and dropout (Jimenez-Murcia et al., 2019). Furthermore, other studies have offered more nuanced classifications of problem gamblers’ game preferences attending to their psychological traits (e.g., impulsivity, delay discounting, punishment and reward sensitivity, and cognitive distortions), relating to two categories (card games, casino games, and skill-based betting versus bingo, slot machines, and lotteries), which partially but not entirely overlap the strategic versus non-strategic dichotomy (Navas et al., 2017). These characteristics do not per se make treatment-seeking online strategic gamblers an entirely novel group, but they arguably transform the profile and magnitude of the gamblers mental health professionals assist in their gambling treatment practices.

Despite the mounting evidence concerning the impact of gambling preferences on multiple areas, the idea of adapting treatment approaches depending on gambling preferences does not appear to have gained any traction. Usually, gambling types (or gambling modes of access) are not typically considered as main predictors of treatment outcomes for gambling disorder (Jiménez-Murcia et al., 2015; Tolchard & Battersby, 2013). Considering the three-factorial understanding of causes that might lead to gambling problems—i.e., individual, situational, and structural factors (Griffiths, 2005)—most research has focused on the individual characteristics of those who develop gambling disorder (e.g., personality, comorbidity, psychological traits, neurological correlates, age, gender), with considerably less attention given to situational and structural determinants.

Arguably, gambling type is fundamentally a structural characteristic because it involves how a gambling product is designed, although it also interacts with other situational factors. This is particularly relevant in the case of online gambling, because the internet has abruptly transformed access, availability, advertising (i.e., situational factors), as well as frequency, duration, and prize structure (i.e., structural factors) of gambling products (McCormack & Griffiths, 2013; Parke & Parke, 2019). Consequently, an examination of how these transformations might also be affecting online problem gamblers and the mental health professionals that treat them is warranted. These issues are even more pressing considering the research that indicates that situational and structural factors are more critical among online rather than offline problem gamblers (Hubert & Griffiths, 2018).

Nonetheless, such an examination does not appear to be underway. Only a minority of studies have examined the influence of gambling preference on treatment outcome (Milton et al., 2002; Sylvain et al., 1997). Perhaps because these early studies—now around two decades old – found no significant differences, this line of research has been largely ignored. A much more recent study reignited the question (Ronzitti et al., 2018) by reporting findings from a large sample of 524 clients seeking help at the National Health Service clinic in the UK. In that study, gambling type differences affecting the treatment outcome were observed, and, more specifically, betting on sports events was found to be a significant predictor of dropping out of treatment. However, these findings are arguably not sufficiently theoretically-informed and need further replication in other settings.

A Canadian study with 32 online problem gamblers demonstrated that the usual cognitive-behavioural therapy (CBT) utilised for land-based problem gamblers was also effective for online problem gamblers (Harris & Mazmanian, 2016a). However, this study did not tackle the potential differences between CBT for land-based versus online gamblers, but between online gamblers undergoing treatment and online gamblers on a wait list. Therefore, no conclusions can be drawn regarding the comparative efficacy and specificities of online gamblers. A follow-up qualitative study by the same authors (Harris & Mazmanian, 2016b) collected open-ended responses from 24 self-identified online gamblers seeking treatment for gambling disorder and also found evidence of CBT being useful for this group but, again, assumed no need to customise CBT for the specific requirements and singularities of online gambling.

In addition to the scarcity of empirical evidence regarding the effects of gambling types or modes of access to gambling on treatment outcomes, the literature exhibits a related gap in reference to how those administering gambling disorder treatment—as opposed to those receiving the treatment—perceive gambling types to influence their practice. Therefore, to the best of our knowledge, the present study is the first to explore the singular challenges that online gambling poses to professionals treating online gamblers and how such challenges differ from the ones posed by traditional offline gamblers. For that purpose, the present research gathered qualitative evidence from mental health clinicians and social workers with experience of dealing with online gamblers, with the aim of outlining the specific barriers for treatment that mental health professionals identify in this particular group of gamblers, which are relatively new, characteristic of them, and not typically shared by traditional problem gamblers. As implied by this objective, the ultimate goals of the study are to collect data concerning the adequacy (or not) of tailoring gambling disorder treatments in accordance with gambling preferences, as well as to help practitioners to adjust to the specificities of online gamblers.

## Methods

### Participants and Procedure

Four focus groups (FGs) were conducted between March and May 2019 in the Spanish cities of Toledo and Madrid (the data were collected before March 2020 and, therefore, the findings were not influenced by the COVID-19 pandemic). The mean duration of the FGs was 77.7 min (SD = 14.5; range = 56 − 97). In total, 28 professionals participated in the discussions: FG1 (n = 8), FG2 (n = 6), FG3 (n = 6), FG4 (n = 8). The mean age for participants was 36.25 years-old (SD = 9.3), and the majority were females (n = 20; 71%). In terms of their occupation, most of them were psychologists (n = 22; 78%), four were social workers (14%), one was a medical doctor (3%), and another one was a social educator (3%). (See Supplementary Table 1 for a detailed account of participants’ characteristics.)

Only two criteria had to be met by individuals to participate in the study: (i) to have experience treating problem gamblers whose preferred mode of gambling was online, (ii) to be involved in the treatment process as a professional with a university degree relevant for problem gambling issues. This second criterion essentially excluded ex-gamblers who volunteer at gambling treatment associations.

The participants were recruited via two separate partners with gambling treatment expertise. Participants from Toledo FGs belonged to different associations across the country devoted to the treatment of problem gambling, all working under the umbrella of the Spanish Federation of Rehabilitated Gamblers (Federacion Española de Jugadores de Azar Rehabilitados [FEJAR]), the largest not-for-profit association problem gambling organisation in Spain. FEJAR hosted an annual two-day seminar in Toledo for problem gambling professionals, and the research team contacted FEJAR to ask for permission to interview them. FEJAR delivered the information to their associates and obtained 14 professionals willing to participate. Two members of the research team travelled to Toledo and carried out two FGs simultaneously. A similar procedure was followed for the Madrid FGs. In this case, the research team recruited the participants through the Union of Associations and Entities for the Attention of Drug Dependencies (Unión de Asociaciones y Entidades de Atención al Drogodependiente [UNAD]), which comprises over 200 associations, with only a small minority dealing exclusively with gambling disorders. Coinciding with a seminar in Madrid, the research team requested permission to have access to professionals with experience in treating online problem gamblers. Fourteen professionals fulfilling the criteria accepted the invitation to participate and the same two research team members conducted simultaneous FGs on site. To the best of the research team’s knowledge, in all four FGs, all the eligible professionals present at the seminars self-selected to participate.

### Ethics and Transparency

The research ethics committee of first author’s university gave formal consent to conduct the study (Ref: ETK-38/18–19). All the participants signed a consent form and were informed about the content and objectives of the study, reassuring them of their right to withdraw from the focus group at any point without any repercussions. The two interviewers hold PhD qualifications, had prior experience in conducting FGs, and disclosed no potential bias to participants. A third research team member was in the room in one of the FGs. The interviews were audiotaped and participants were given a complimentary gift at the end of the FGs. They were not contacted for comment or feedback once the data were analysed. Because the study dealt qualitatively with health-related issues, the data reported here were structured according to the 32-item checklist contained in the Consolidated Criteria for Reporting Qualitative Research (COREQ; Tong et al., 2007). The data reported here is part of a larger project based on FGs interviews with 68 participants comprising both problem gamblers undergoing treatment and treatment professionals concerning the effects of online gambling.

### Data Analysis

The FG discussions were semi-structured according to a predetermined script but focus group leaders had room for improvisation if interesting topics and exchanges emerged during conversations. The script had four main blocks, and all referred to the differences between online and offline gamblers in treatment: (1) profile differences (e.g., age, debt, motivation, education), (2) barriers for access to treatment: (2.1) self-awareness, (2.2) how they asked for help, (2.3) self-concept and self-perception about online gambling; (3) barriers during treatment: (3.1) cognitive aspects (skill-based gambling-related cognitions, fixations and beliefs), (3.2) group dynamics (interaction with offline gamblers, challenges associated with being younger); and (4) gender differences (not reported in the present study).

The collected data were transcribed verbatim by a graduate school student who was paid for the task. Two independent coders from the research team analysed the entire dataset using NVivo 11, and a third team member offered her input on those instances where coders disagreed. The theoretical underpinnings for the analysis were those of the thematic analysis in qualitative research (Braun & Clarke, 2006; Wilkinson, 2004). The underlying analytical approach was to examine the data in an inductive manner, without necessarily replicating the preconceived blocks outlined in the script. To substantiate this purpose, three main coding cycles were carried out (Saldaña, 2009). First, an open coding cycle (*holistic coding*) was conducted to identify a preliminary list of nodes (i.e., themes) that could best condense the insights of participants. These findings were shared and discussed between the two coders, and gave way to a second round of coding (*structural coding*) characterised by a more systematic way of examining the data and merging preliminary nodes into larger themes. Finally, the resulting themes were further classified and hierarchised to produce a single, all-encompassing interpretation of the singularities of online gambling in treatment (*pattern coding*).

In total, 1023 references were coded in the first cycle, resulting in 58 preliminary nodes (see Supplementary Table 2). These were further reduced to 10, which were the ones relevant for the present study. These nodes were expanded again to create subcategories in order to build a detailed classification.

## Results

One of the dynamics in the FGs had to do with describing the profile of online gamblers as opposed to offline gamblers. Forty-four references to online gambler characteristics were mentioned by participants (henceforth, treatment providers, for clarity). After merging several nodes, a condensed version of the most mentioned attributes emerged: (i) being of younger age, (ii) lack of conflicts at home and at work/educational centre, rarely presenting violent or aggressive behaviour, (iii) as a consequence, their gambling disorder is only being identified by overdue debt, (iv) co-occurring conditions with technology-related abuse rather than other substance-related addictions, and (v) skill-based gambling. Some treatment providers mentioned that those who were fundamentally offline gamblers but felt the need to transition to online gambling at some point (e.g., because they wanted to escape scrutiny once their gambling increased or because they have been caught in their usual gambling environments) did not conform to these profile characteristics but treatment providers questioned whether they could be considered online gamblers as such.

### Being of Younger Age

Many treatment providers noted that some of the differences between offline and online gamblers were not as associated to the gambling modality but to the age of those who engaged in it. Online gamblers, inasmuch as young gamblers, exhibit a number of characteristics that are fundamentally related to the risks of adolescence. In this regard, the treatment providers identified a series of attributes encompassed in being a young online gambler:

1. The first of these concerned lower self-awareness, lower motivation to change, and lower adherence to treatment. In general, treatment providers thought that, although gamblers of any kind tend to exhibit low problem gambling awareness and are usually forced by family members to seek help, young online gamblers accentuate such tendency, with very low motivation to engage in treatment activities and a high probability of abandoning it.

Some treatment providers mentioned that being able to experience in first person the negative consequences of excessive gambling was a driver of self-awareness and motivation. Older gamblers usually suffer consequences across numerous life aspects including family, work, friends, and health. In some treatment providers’ views, younger gamblers do not necessarily experience consequences the same way older people do, and the fact that sometimes young online gamblers solely suffer monetary effects makes them unaware of the overall impact gambling has had on their lives:

*I think they [skill-based gamblers] have less problem gambling self-awareness, they’re different from land-based gamblers. I’m thinking about this guy we have in our association. He came once, he left, and came back with twice the debt he had the first time. Today, more than a year later, he still doesn’t see gambling is his problem. He thinks the lack of money is his problem. He doesn’t realise that is the consequence [emphasis in the original] of his problem, not the problem itself. And we’re not making progress because we’re unable to make him see that* (P41, 43 years, male psychologist).

(2) One of the things that separates traditional offline gamblers and younger online ones is their compliance with the therapeutic goal. In most cognitive-behavioural psychology treatments for gambling disorder, abstinence is the non-negotiable ultimate goal. Recovering any sense of control over one’s gambling behaviour once the disorder has started is deemed delusional. According to treatment providers, gamblers of all types struggle to adjust to the idea that gambling will never be a suitable activity for them, and that complete abstinence will be required even after finishing treatment. However, younger gamblers are particularly belligerent to this idea. As one treatment provider explains (P44, 37 years, female psychologist), the notion of *“never gambling again”* has very different meanings for different age groups, for two main reasons. First, for a person in their 50 s or 60 s the actual duration of the term ‘never’ is shorter than for somebody in their early 20 s. This is, objectively speaking, a briefer commitment as people get older. Second, and most importantly, older gamblers have usually tested themselves and relapsed, and are therefore, more convinced about the necessity of a full abstinence. Younger gamblers sometimes are yet to experience their first relapses, which make them more vulnerable to resuming gambling because they want to test if they have acquired self-control tools during therapy, as this excerpt illustrates:
  *The thing with young people is that when they finish treatment, they’ve been for some time without playing and have acquired self-control abilities, they start wondering ‘why can’t I gamble from time to time like everyone else?’ And then they challenge themselves and that’s basically one of the most frequent reasons for a relapse* (P66, female social worker).
(3) Treatment providers typically reported having pre-established protocols in their treatment centres involving strict rules for those in treatment. For example, most of the treatment providers reported that self-excluding from gambling was a prerequisite to begin therapy. However, many treatment providers appeared to be in agreement that, while these fixed rules were very rarely ignored with traditional offline gamblers, that was not the case with young online gamblers. One of the things that most treatment providers agreed upon was the need for negotiation with young people, the plasticity to bend the rules to accommodate the psychology of online gamblers to avoid low adherence to treatment.

### Lack of Social Conflict

Another area of agreement among most treatment providers concerned the relatively lower social conflicts that younger gamblers present in comparison to offline gamblers. In the treatment providers’ opinion, online gamblers tend to have no previous record of antisocial behaviour, and are less likely to resort to violence. Even in those cases where antisocial behaviour has occurred, it is usually less visible (e.g., they steal within the family circle involving no violence) and raises fewer social alarms. Among online gamblers, all the problems appear to be contained within gambling behaviour, with few or no co-occurring conflicts. In such scenarios, treatment providers mentioned that it is very hard as professionals to argue in favour of restricting the gambler’s life beyond their gambling, because no other area appears to be negatively interacting in a visible way. This includes limiting alcohol intake, mobile phone use, or stopping seeing friends who continue gambling. Although one of the tenets of cognitive-behavioural therapy is reducing the exposition to cues that might trigger gambling craving, online gamblers were said to be particularly reluctant (relative to offline gamblers) to cutting down any of these activities, especially when they show no problematic pattern with them (e.g., problem drinking).

### Co-occurring Technology Abuse

A major problem treatment providers encountered when they treat online gamblers is finding viable alternatives to gambling. Online gamblers, as opposed to offline gamblers, appear to have a smaller repertoire of alternative activities they can fill their time with. It is especially troublesome that most of such alternatives revolve around virtual activities within the digital world, as this treatment provider explained:

*Somebody else, older or with a partner, would tell you they are enjoying new things now, things they didn’t enjoy before, practicing sport… They feel good. They see a response and they keep working. Adolescents don’t see that. They don’t see a reward and they don’t keep working. For me, it’s much harder to work [with online rather than traditional gamblers] on alternative leisure activities, how to manage free time, communication skills, prevention…Online gamblers are tough because there is nothing else for them, everything is connected to new technologies. I cannot recommend them to go hiking, practicing sports* (P59, 46 years, female social worker).

The fact that online gambling might many times be intermingled with other technology-based activities makes it harder for the professionals here to disentangle maladaptive online consumptions from perfectly adequate ones.

### Debt

Most of the treatment providers pointed out that online gamblers tend to incur higher debt than offline gamblers, and over a shorter period of gambling disorder development. For some treatment providers, how debt is handled is one of the defining characteristics that distinguish online from offline gamblers. Because online gamblers are generally younger, sometimes they are not economically independent yet (i.e., they are studying and/or working part-time). This means that when their gambling generates debt, their parents will try to pay it off on their behalf. Many treatment providers were emphatic regarding how often this situation emerged and how negative it was for the recovery of gamblers. First, treatment providers were unanimous in saying that older gamblers suffer greatly from their debts and sometimes struggle to keep up with loan terms. But that suffering helps them experience very vividly the consequences of their gambling and is a driver for avoiding relapse. Second, many times debt is the only negative consequence younger online gamblers will face and taking it away from them might be counterproductive, as one treatment provider reflects:

*think it’s harder for them [young online gamblers]. Because they see older people, and they’ve lost their family, their job, and they’re having a really tough time, but they [younger people] cannot relate to that experience. They’ve basically lost nothing* (P52, 29 years, female psychologist).

This treatment provider summarises the effects of allowing parents or other family members becoming accountable for a gambler’s debt, and points to the fact that, for a recovering gambler, concluding that *“I’ve lost nothing”* only a few months after engaging in a gambling spree teaches the wrong lesson. In her opinion, paying one’s debt is essential not only because it is part of growing up and becoming a responsible adult, but also because paying back money that is owed usually takes time, sometimes years, and that long process ensures that in years to come, young gamblers will feel as current something they did long time ago, which keeps them away from relapse. It was also noted that online gamblers appear to resort more often than offline gamblers to online payday loans and credit card debt to finance their gambling.

### Skill-Based Gambling

Younger generations tend to have higher education, and many treatment providers noted that among online gamblers there is a larger proportion of university students than among offline gamblers. For some treatment providers this translated into a greater facility to communicate with online gamblers because they appear to have learned more cognitive skills to use in therapy. Conversely, such cognitive skills can also work against the therapeutic goals in some contexts, as in the case of cognitive restructuring in cognitive-behavioural therapy. A few treatment providers specifically mentioned how actively young online gamblers resisted therapists’ arguments and conceptions about online gambling.

One aspect of online gambling that all of the treatment providers agreed upon had to with how online gambling products work. All of them either felt insecure about the actual mechanics of gambling online or dismissed such knowledge as irrelevant for their task as mental health professionals, but all acknowledged some degree of ignorance. In this regard, a few treatment providers, as in the example below, show erroneous ideas about online gambling:

P41 (43 years, male psychologist): *We need more information about the ins and outs [of online gambling] because we’re in diapers about if people can win or not … I seriously doubt they win but…*

P45 (28 years, female psychologist): *… there are only a few things in life that are 100% chance. You can always do something, even in bingo, which is [based on] chance. If I buy 20 cards, I’m going to have a little bit more chance of winning than if I just buy one.*

In the example, treatment provider P45 verbalises a common mistake about the mathematical probability in games of chance. In bingo, the longer a gambler plays the more likely their outcomes will reflect the implicit probability built in the bingo product by the gambling provider. Naturally, such probability does not increasingly become more beneficial for gamblers over time; quite the opposite. Besides this example, most of the treatment providers’ verbalisations about gambling were correct but mostly applied to traditional only chance-based gambling products (i.e., lottery, electronic gaming machines, bingo, roulette), but were not readily applicable to mixed, skill-based gambling forms. However, none had given serious thought to the idea that newer online gambling products might affect the way therapists’ approach to cognitive restructuring. In general, treatment providers reproduced in their clinical settings the way distorted cognitions have been traditionally addressed, for example, explaining how the gambler’s fallacy works, but when confronted about the timeliness and applicability of these conceptualisations in the context of skill-based online gambling products, they were more hesitant about it.

To add to the confusion, in some treatment providers’ verbal recollections, a number of past patients who engaged in online sports betting and poker were remembered as professional gamblers. The figure of the professional poker player or sports bettor (including tipsters) who seeks treatment for gambling disorder was confusing for many treatment providers. Health professionals are sceptical about the existence of treatment-seeking gamblers who are self-proclaimed professionals. With traditional purely chance-based gamblers, accounts of systematic winnings were deconstrued as brief streaks of good luck, distorted cognitions, or, simply, lies. But with games that combine chance and skill, the consideration of gamblers who present themselves as professionals becomes more ambiguous.

## Discussion

The present study provides evidence regarding the specific challenges that treatment-seeking online gamblers pose to mental health professionals. It collected data from group interviews with social workers, psychologists, and a medical doctor about the difficulties they perceive they face when dealing with online gamblers, as compared to their experience with more traditional offline gamblers. The study found evidence of the existence of differences between these two groups of gamblers, making the treatment of online gamblers a substantially singular experience that needs specific adjustment from traditional problem gambling therapy in a number of facets, as detailed below.

Treatment providers reported issues associated with the young age of online gamblers. There is consistent empirical evidence from many problem gambling treatment contexts that online gamblers seeking treatment are significantly younger than offline gamblers (Edgren et al., 2017; Estévez et al., 2017; Griffiths et al., 2009; Lloyd et al., 2010). However, relatively less attention has been paid to what it means for gambling disorder treatment to have such a growing pool of young online gamblers. The findings from the present study suggest that online gamblers might be *too* protected from the negative consequences of their excessive gambling. The fact that they were, generally speaking, surrounded by a supporting network of family members was arguably beneficial in many aspects of their recovery, but also, counterintuitively, removed some of the effects of their gambling that might help them mature and be less vulnerable to relapse. This was exemplified by the concerns of some mental health workers that young online gamblers only perceived the monetary impact of their gambling, but ignored other social, emotional, or trust-related consequences of it. A more biopsychosocially grounded approach to gambling disorder, making online gamblers more aware of the whole spectrum of harm that gambling has caused in them and those around them (Browne et al., 2017; Li et al., 2017) appears a reasonable way of tackling this issue.

A similar preoccupation the study found has to do with debt payment. The findings provide evidence of the negative long-term consequences of family members paying off young online gamblers’ debt. This finding has to be interpreted cautiously. Debt is generally larger among online than offline gamblers (Gainsbury et al., 2014; Mihaylova et al., 2013), with financial institutions increasingly under scrutiny because of their passivity to pre-emptively identify problematic gambling patterns (Swanton & Gainsbury, 2020; Swanton et al., 2019). Debt size affects gambling behaviour (Crewe-Brown et al., 2014), and gamblers who find themselves unable to meet their financial obligations consequently experience higher psychological distress (Oksanen et al., 2018). Therefore, suggesting family members to withdraw from helping their loved ones to overcome their financial problems so they can learn their lesson might be less effective than committing to some sort of intermediate arrangement, wherein debt pressure is more sustainable while the recovering gambler is still responsible for their gambling consequences. The effects of family members ‘bailing-out’ young gamblers has already been identified in the literature as a potential deterrent of adolescent enrolment in gambling treatment programmes (Chevalier & Griffiths, 2004).

Two of the ideas that treatment providers emphasised most during the interviews was the necessity to negotiate beyond rigid rules when dealing with young online gamblers, and the meaning and real duration that abstinence has for them as opposed to older gamblers. Although none of the treatment providers explicitly mentioned it, these related findings might spur the debate about the potential adequacy of combining abstinence and controlled gambling goals in gambling therapy. In therapy for gambling disorder in Spain and elsewhere, abstinence is widely accepted to be a prerequisite for improvement. However, numerous studies have demonstrated that self-selected therapy goals of moderate or controlled gambling might be equally effective in treating gambling disorder (e.g., Blaszczynski et al., 1991; Dowling et al., 2009; Mazar & Volberg, 2019; Slutske et al., 2010; Stea et al., 2015). Taking into account the (i) low access and adherence to treatment, and (ii) high impact on socialisation, negotiating therapeutic goals with young online gamblers to include some versions of moderate (‘controlled’) gambling might be worth consideration.

The online gambler profile discussed in this paper—characterised by rare antisocial behaviour and quick and large debt—is difficult to identify and departs from previous characterisations of what a problem gambler looks like. Online gamblers do not bear some of the social stigmas traditionally attached to gamblers (Lang & Rosenberg, 2017), and in the particular case of online sports bettors, the most represented group among online gamblers in Spain, a positive social perception might be operating in the opposite direction, reducing the stigma that tarnishes gamblers but, paradoxically, also protecting them from engaging the behaviour (Lopez-Gonzalez et al., 2018a). To counteract such reduced public visibility, online behavioural tracking tools appear to be an adequate alternative to identify problematic gambling (Chagas & Gomes, 2017; Griffiths, 2019).

The findings from the present study are inconclusive regarding the adequacy of cutting down or prohibiting the use of non-gambling related internet activities (e.g., using social media, playing online videogames) as a pre-requisite to improve online gambling behaviour. Although the difficulty of providing non-digital viable alternatives for online gambling due to technological overdependence has been mentioned, the advantages and efficacy of taking such measures is unclear.

Finally, the skill components of the most popular online gambling forms are a matter of concern. The treatment providers’ insights indicated that an effective therapy for online gamblers must consider the specific configuration of newer online gambling products, raising awareness among mental health workers about their ins and outs. Traditional ideas concerning how randomness works in slot machines, roulette, or lotteries might no longer be applicable in the exact same form, and the failure to acknowledge it when engaging in conversations with online gamblers might be detrimental to the therapeutic alliance. This is especially relevant when dealing with distorted cognitions in cognitive-behavioural therapy, considering how products such as online sports betting cater to particular manifestations of the illusion of control (Lopez-Gonzalez et al., 2018b).

The present study includes some limitations. First, in the composition of Spanish online gamblers seeking for help, sports bettors were overrepresented to the detriment of other online gambling forms. In these focus groups, most mental health workers had online sports bettors in mind when talking about online gamblers, since this is the group they have become more familiarised with in their daily practice. Therefore, the generalisation of these results may be difficult in other countries where the predominant online gambling types are different. Second, the inductive method of data interpretation made the findings emerge once the interviews were conducted. As the study design did not anticipate reaching treatment providers for feedback, the researchers are unclear with regard to how they might feel about the conclusions and recommendations outlined in this paper, especially concerning controlled versus abstinence-based approaches for online gamblers.

## Conclusion

This study is one of the first to provide evidence concerning the perceived challenges for mental health workers of the new profiles of online gamblers. The study contended that online gamblers not only present differing characteristics from those engaging in traditional offline gambling forms, but also that online gambling itself, as a specific gambling form with singular situational and structural characteristics, presents significant differences that gambling disorder treatment should consider to better tailor its therapeutic goals. The paper departs from the ‘individual factors’ approach that tries to categorise disordered gamblers as individuals with a propensity to endorse (or not) specific features that makes them more susceptible to experience gambling problems, and instead explores the interaction between such individuals and the distinctive characteristics of online gambling as a product. In conclusion, the paper acknowledges that the internet has transformed the provision and design of gambling, and has consequently affected the way gamblers interact with online gambling products. Assuming this scenario, such interaction produces specific problems for mental health professionals accustomed to treating traditional offline gamblers, and they perceive their therapy must adapt to them. All things considered, the paper makes the case for the convenience of tailoring gambling disorder treatments in accordance to gambling preferences, and training mental health professionals on the specificities of evolving gambling products.

## Supplementary Information

Below is the link to the electronic supplementary material.

Supplementary file1 (DOCX 109 KB)

Supplementary file2 (DOCX 73 KB)

## Data Availability

Supplementary tables are included in the submission. The consent form that participants signed did not include the publication of the raw data (interview transcripts or audios). These materials will not be openly available.
