# Supplementary material for: Do Online Gambling Products Require Traditional Therapy for Gambling Disorder to Change? Evidence from Focus Group Interviews with Mental Health Professionals Treating Online Gamblers
Source: J Gambl Stud. 2021 Oct 16;38(2):681–97. doi: 10.1007/s10899-021-10064-9 (PMC8520338; doi:10.1007/s10899-021-10064-9)
Supplement: Supplementary file 1 — Supplementary file1 (DOCX 109 KB) [file 10899_2021_10064_MOESM1_ESM.docx]

Table 1. Detailed list of mental health professionals

| Code | City | Age | Sex | Occupation |
| --- | --- | --- | --- | --- |
| P41 | Toledo (1) | 43 | Male | Psychologist |
| P42 | Toledo (1) | 44 | Male | Psychologist |
| P43 | Toledo (1) | 29 | Female | Psychologist |
| P44 | Toledo (1) | 37 | Female | Psychologist |
| P45 | Toledo (1) | 28 | Female | Psychologist |
| P46 | Toledo (1) | 29 | Male | Psychologist |
| P47 | Toledo (1) | 30 | Female | Psychologist |
| P48 | Toledo (1) | 26 | Female | Psychologist |
| P49 | Toledo (2) | 29 | Female | Social worker |
| P50 | Toledo (2) | 27 | Female | Psychologist |
| P51 | Toledo (2) | 28 | Female | Psychologist |
| P52 | Toledo (2) | 29 | Female | Psychologist |
| P53 | Toledo (2) | 37 | Male | Medical doctor |
| P54 | Toledo (2) | 58 | Female | Psychologist |
| P55 | Madrid (1) | 32 | Male | Psychologist |
| P56 | Madrid (1) | 44 | Male | Psychologist |
| P57 | Madrid (1) | 42 | Female | Psychologist |
| P58 | Madrid (1) | 32 | Female | Social educator |
| P59 | Madrid (1) | 46 | Female | Social worker |
| P60 | Madrid (1) | 55 | Female | Psychologist |
| P61 | Madrid (2) | - | Female | Psychologist |
| P62 | Madrid (2) | - | Female | Psychologist |
| P63 | Madrid (2) | - | Female | Psychologist |
| P64 | Madrid (2) | - | Male | Psychologist |
| P65 | Madrid (2) | - | Female | Social worker |
| P66 | Madrid (2) | - | Female | Social worker |
| P67 | Madrid (2) | - | Male | Psychologist |
| P68 | Madrid (2) | - | Female | Psychologist |

*Notes*. Focus groups Toledo (1) & Toledo (2) took place on March 31, 2019, simultaneously; Madrid (1) & Madrid (2) on May 27, 2019, simultaneously. Age data from Madrid (2) was lost due to a recorder malfunction.
