# Supplementary material for: Do Online Gambling Products Require Traditional Therapy for Gambling Disorder to Change? Evidence from Focus Group Interviews with Mental Health Professionals Treating Online Gamblers
Source: J Gambl Stud. 2021 Oct 16;38(2):681–97. doi: 10.1007/s10899-021-10064-9 (PMC8520338; doi:10.1007/s10899-021-10064-9)
Supplement: Supplementary file 2 — Supplementary file2 (DOCX 73 KB) [file 10899_2021_10064_MOESM2_ESM.docx]

Supplemental Table 2. List of nodes and number of references for the preliminary holistic coding

| Node name | Reference |
| --- | --- |
| Appeals of online gambling | 15 |
| Big bets=online | 2 |
| Bonuses | 3 |
| Bragging rights | 27 |
| Cashing out | 16 |
| Cognitions about gambling | 38 |
| Descriptors of online gambling proliferation | 9 |
| Differences online-offline gambler | 64 |
| Errors about gambling in therapists | 4 |
| Features of online gambling | 43 |
| Gamblers | 7 |
| Gambling at work | 54 |
| Gender diff Encouragement by partner | 9 |
| Gender diff in gambling behaviour | 8 |
| Gender differences | 1 |
| Hard to stripe gambling ideas | 37 |
| Individual device | 5 |
| Lies to family | 19 |
| Match fixing | 8 |
| Mental health | 7 |
| Mistakes when using the mobile to gamble | 5 |
| Mobile phone as indicative of addiction | 3 |
| Money withdrawals online | 3 |
| Never gambling again | 17 |
| Notifications | 5 |
| Online customer service | 19 |
| Online device | 27 |
| Online money | 32 |
| Online-offline balance | 22 |
| Overcoming online prohibition | 20 |
| Personal factor | 4 |
| Professionals | 6 |
| psychologists aware of online gambling | 27 |
| Responsible gambling measures | 51 |
| Role games | 1 |
| Skill in online gambling | 59 |
| Sleep deprivation | 7 |
| Socializing | 8 |
| Sportspeople gambling | 3 |
| Stopping mobile gambling=quiting mobile phone | 12 |
| Tennis | 7 |
| Tipster eluding problem gambling | 1 |
| Tipsters | 53 |
| Treatment-Access to therapy | 9 |
| Treatment-Debts | 5 |
| Treatment-difficulties in therapy | 47 |
| Treatment-gamblers turned pro | 5 |
| Treatment-Immigrants | 1 |
| Treatment-low risk perception | 6 |
| Treatment-profile of online gambler | 44 |
| Treatment-Socialization | 14 |
| Treatment-Stimuli control | 8 |
| Treatment-Support of family | 3 |
| Videogames-micropayments | 4 |
| WhatssApp & Telegram | 12 |
| Winning money with sports betting | 20 |
| Women gambling | 57 |
| Workers gambling | 10 |
| **Total nodes = 58** | **Total references = 1023** |
